# Determinants of fruit and vegetable consumption among children and adolescents: a review of the literature. Part II: qualitative studies

**DOI:** 10.1186/1479-5868-8-112

**Published:** 2011-10-14

**Authors:** Rikke Krølner, Mette Rasmussen, Johannes Brug, Knut-Inge Klepp, Marianne Wind, Pernille Due

**Affiliations:** 1 National Institute of Public Health, University of Southern Denmark, Copenhagen, Denmark; 2 Department of Epidemiology and Biostatistics and the EMGO Institute for Health and Care research, VU University Medical Center, Amsterdam, The Netherlands; 3 Department of Nutrition, Faculty of Medicine, University of Oslo, Norway; 4 GGD Gelre-IJssel (Community Health Service), Apeldoorn, The Netherlands

**Keywords:** fruit, vegetables, child, adolescent, qualitative, review, school, family, availability, eating behaviour

## Abstract

**Background:**

Large proportions of children do not fulfil the World Health Organization recommendation of eating at least 400 grams of fruit and vegetables (FV) per day. To promote an increased FV intake among children it is important to identify factors which influence their consumption. Both qualitative and quantitative studies are needed. Earlier reviews have analysed evidence from quantitative studies. The aim of this paper is to present a systematic review of qualitative studies of determinants of children's FV intake.

**Methods:**

Relevant studies were identified by searching Anthropology Plus, Cinahl, CSA illumine, Embase, International Bibliography of the Social Sciences, Medline, PsycINFO, and Web of Science using combinations of synonyms for FV intake, children/adolescents and qualitative methods as search terms. The literature search was completed by December 1st 2010. Papers were included if they applied qualitative methods to investigate 6-18-year-olds' perceptions of factors influencing their FV consumption. Quantitative studies, review studies, studies reported in other languages than English, and non-peer reviewed or unpublished manuscripts were excluded. The papers were reviewed systematically using standardised templates for summary of papers, quality assessment, and synthesis of findings across papers.

**Results:**

The review included 31 studies, mostly based on US populations and focus group discussions. The synthesis identified the following potential determinants for FV intake which supplement the quantitative knowledge base: Time costs; lack of taste guarantee; satiety value; appropriate time/occasions/settings for eating FV; sensory and physical aspects; variety, visibility, methods of preparation; access to unhealthy food; the symbolic value of food for image, gender identity and social interaction with peers; short term outcome expectancies.

**Conclusions:**

The review highlights numerous potential determinants which have not been investigated thoroughly in quantitative studies. Future large scale quantitative studies should attempt to quantify the importance of these factors. Further, mechanisms behind gender, age and socioeconomic differences in FV consumption are proposed which should be tested quantitatively in order to better tailor interventions to vulnerable groups. Finally, the review provides input to the conceptualisation and measurements of concepts (i.e. peer influence, availability in schools) which may refine survey instruments and theoretical frameworks concerning eating behaviours.

## Background

Epidemiological evidence for the health benefits of a diet rich in fruit and vegetables is substantial [1-3]. Despite this fact large proportions of children and adolescents do not meet the World Health Organization goal of a daily intake of at least 400 grams of fruit and vegetables [4-6]. Longitudinal studies suggest that eating behaviour such as fruit and vegetable consumption tracks into adulthood which points at the importance of establishing healthy eating behaviour among children and adolescents [7-9].

To enable the development of relevant, effective fruit and vegetable promoting intervention programs and policies targeting children and adolescents it is important to identify the various factors which may influence their consumption of fruit and vegetables and both qualitative and quantitative studies are needed [10].

Quantitative studies are needed to quantify and rank the importance of determinants for children's fruit and vegetable consumption and for example, to assess sociodemographic variations in these. In the first part of this review the evidence from 98 quantitative studies of fruit and vegetable intake among children and adolescents was analysed [11]. In conclusion, the determinants for high consumption levels of fruit and vegetable supported by the strongest evidence were female gender, low age, high socioeconomic position (SEP), high preferences for fruit and vegetables, high parental intake of fruit and vegetables and high availability/accessibility of fruit and vegetables at home.

Qualitative studies can add to this knowledge in several ways. They provide the opportunity to identify yet unknown factors as the research techniques give room for unprecedented answers as opposed to the highly structured interviews used in surveys. Qualitative studies can thereby contribute to the development of comprehensive survey instruments and generate hypotheses about associations which can be tested in future quantitative studies. Furthermore, qualitative studies can generate a more thorough understanding of fruit and vegetable consumption as they usually aim at reflecting the diversity of views on the studied phenomenon within a given population [10,12]. Finally, qualitative methods are a useful tool within formative research aiming at designing effective interventions tailored to a given population's own needs and contextual conditions.

Systematic reviews are important for evidence-based practice. Such review efforts have almost solely been focused on quantitative studies which is also the case for reviews concerning dietary behaviours [10,11,13-15]. It is important also to review the qualitative research to increase insight into processes which influence young people's fruit and vegetable intake. Thus, the aim of the present paper is to present part two of a systematic review of peer-reviewed papers, this time qualitative studies of 6-18-year-olds' views and experiences regarding determinants of their intake of fruit and vegetables.

## Methods

### Literature search strategy

We conducted a comprehensive systematic and exhaustive literature search of the following electronic databases from the year of their inception to December 1st 2010: Anthropology Plus (1900 onwards), CSA illumine [ERIC (1966 onwards), Econlit (1969 onwards), Sociological abstracts (1952 onwards), Social Services abstracts (1979 onwards), Worldwide political Science abstracts (1975 and onwards)], Cinahl (1981 onwards), Embase (1980 onwards), International Bibliography of the Social Sciences (1981 onwards), PsycINFO (1806 onwards), Pubmed Medline (1966 onwards), Web of Science [Science Quotation Index expanded (1945 onwards), Social Sciences Quotation Index (1956 onwards), Art & Humanities Quotation Index (1945 onwards)]. Together these databases covered a variety of disciplines: anthropology, economics, health education, medicine, nursing research, nutrition, political science, psychology, social care, and sociology.

## Search terms

We applied the same electronic search strategy in all databases. Peer-reviewed publications were searched by combining the following search terms:

1) [(fruit OR fruits OR vegetable OR vegetables) OR ((diet OR diets OR nutrition OR eating OR food) AND (healthy OR healthful))]

AND

2) [child OR children OR childhood OR adolescent OR adolescents OR adolescence OR youth OR young OR teen OR teens OR teenager OR teenagers OR student OR students OR girl OR girls OR boy OR boys OR pupil OR pupils OR schoolchild OR schoolchildren]

AND

3) [anthropology OR anthropologic OR anthropological OR ethnography or ethnographic or ethnographical OR qualitative OR focus group OR focus groups OR grounded theory]

The literature search was completed by December 1st 2010 and yielded 2, 813 records. Titles and abstracts were systematically screened and considered for inclusion by one reviewer (RK) according to pre-specified inclusion and exclusion criteria. Papers were included if they 1) investigated determinants of fruit and/or vegetable intake, either as the primary focus or as part of healthy eating (diet, nutrition or food) where information specifically related to fruit and/or vegetables could be identified, 2) explored children's and adolescents' own views and experiences of motivators and barriers to eating fruit and vegetables, 3) were based on qualitative research methods (data collection and analysis), and 4) were based on populations within an identifiable age-range of 6 to 18 years (school-aged children). We applied a broad definition of qualitative research methods. Qualitative data collection methods were defined to include observations, face to face interviews, focus group discussions, and action research. Similarly qualitative data analysis was not restricted to certain analytical traditions but defined to include various approaches such as grounded theory, comparative methods, phenomenological analysis, and content analysis.

Papers were excluded if they were 1) based on quantitative research methods (data collection or analysis), 2) review studies, 3) non-peer reviewed or unpublished manuscripts (abstracts or dissertations), and 4) reported in languages other than English.

If the eligibility of a paper was questionable, the paper was provisionally included. Through this screening process 248 potentially relevant papers were identified and the full-text articles were read thoroughly and considered for inclusion by at least two reviewers. If the two authors did not agree upon inclusion, a third author was asked for her opinion, and arguments for eligibility were discussed to come to a joint decision. We also checked our own records, reference lists of all eligible papers as well as bibliographies of existing reviews for relevant papers. Only 24 additional records were identified through these sources, of which one was included in the present review [16].

### Analytical approach

The review of the papers we included followed a 3-step protocol and was based on published recommendations and other systematic reviews of qualitative research as well as the protocol applied in the earlier review from this research group [11,17-20]. We developed standardised templates which were used in each step. All papers were reviewed independently by at least two of the authors (RK and PD/MR). Inter-reviewer disagreement in relation to data extraction was solved by discussion until consensus on interpretation was reached or acknowledged as different perspectives to refine the coding scheme. Two of the three reviewers had conducted qualitative research themselves (RK/PD). All reviewers were involved in the earlier review of quantitative studies (part one) [11] and are researchers within the area of adolescent health and health behaviour.

## Step I: Data extraction

In the first step, we extracted data from each paper into separate paper-specific summary forms on the following topics: 1. Country setting, 2. Aims, 3. Study population/sample characteristics, 4. Study design, 5. Preconceptions (e.g. theoretical framework), 6. Phenomenon of interest (e.g. fruit and vegetable intake as primary focus or as part of healthy eating), 7. Use of interview guide, 8. Analysis, 9. Main topics (For papers on healthy eating, findings were only extracted if specifically related to fruit and vegetable intake), 10. Main conclusions/discussion, 11. Study limitations, and 12. Validity issues.

## Step II: Quality assessment

The second analytical step included a systematic assessment of the internal and external validity and overall methodological quality of each paper. We applied a list of quality criteria (see additional file 1 for more information) suggested by different papers on qualitative methodology [17,21,22] and mapped methodological strengths and limitations for each study in a quality assessment scheme. We assessed whether 1) the relevant information on methodological issues/selected criteria was provided in a clear, explicit and sufficient way and if we were able to follow the analysis and the transformation of raw data (interviews) to findings/themes (*high communicative validity*) 2) whether the quality of craftsmanship in the investigation was high so the findings appeared to be credible, 3) whether the transferability of findings was discussed (high external validity) and 4) if studies could guide future research and practice (pragmatic validity) [23]. At least two of the authors assessed the quality of the papers independently (RK and MR/PD). Inconsistencies were discussed until consensus was reached. If consensus was hard to obtain the opinion of the third author was sought or a second opinion was obtained from an impartial colleague, a professor experienced in qualitative research methods and working with children as respondents (peer debriefing). We applied two measures for overall quality of the paper, a count of quality criteria met and the evaluators' overall assessment of the quality of the paper.

## Step III: Synthesis of findings

The third step was extraction of all key findings and illustrative quotations into one comprehensive overall evidence scheme. We categorised the findings by constant comparative analysis - a systematic procedure to identify similarities and differences in findings across studies [17,24]. Findings from different studies were grouped into the same theme if the analysis showed they shared common characteristics or represented different aspects/dimensions of the same theme. Dissimilar findings were separated out and renamed into other themes. The potential of qualitative research is to depict the scope and variation in children's views and extreme or unusual views mentioned by a few informants might represent important properties of a key theme [22]. Therefore findings mentioned only by a few informants or by a few studies were also considered relevant in this review. By this conceptual analysis we de-contextualised our findings in order to identify common themes/potential determinants across countries. To ensure that context was not lost in the synthesis we kept track of the country origin of each extracted key finding and quotation to be able to highlight potential country-differences in the presentation of results. The results from all studies meeting the inclusion criteria were summarized irrespective of methodological quality. As part of sensitivity analysis, papers of high and low methodological quality were compared for congruence and variation in results. We also assessed if different conclusions were reached if the studies of low quality were excluded from the synthesis.

## Results

### I. Study selection and study populations

Thirty-one papers matched all inclusion criteria and were included in the review. The papers have been published from 1973 to 2009. Most studies were conducted in the United States of America (US, N = 19), followed by Europe (N = 7). Thirteen studies used source triangulation and interviewed both schoolchildren, parents, school staff or key informants from schools or communities. Almost all studies recruited participating children through schools. The majority of studies included general adolescent populations but three studies focused on a selected sample considered to be at greater risk of unhealthy dietary habits such as alternative high school students or diabetic youth. The studies included covered an age range from four to 19 years of age. All studies except one study of boy scouts [25] and four with no information on gender, included both boys and girls. Table 1 summarises the study characteristics.

**Table 1:** Study characteristics

Study by first author [Ref. ID]	Country setting	Pheno-menon of interest (outcome)	Sampling and participants	Child socio-demographics: **A: sex. B: age**. **C: school year. D: SEP. E: ethnic background**.	Data collection methods and no. of FGs or interviews	Theoretical framework	Analytical method/approach	Main topics related to FV intake
**Baranow-ski et al. 1993 **[29]	US	FV intake	School-based (1 school): 235 schoolchildren, 15 parents, 8 teachers, 4 school food service workers.	A: no info. B: no info. C: 4 & 5. D: pre-dominantly lower SEP. E: more than 50% Afro-Americans and the rest mostly Anglo-Americans.	FG discussions: 5 year 4 schoolchild FGs, 5 year 5 schoolchild FGs, 2 parent FGs, 2 teacher FGs, 1 school food service worker FG.	Social cognitive theory: reciprocal determinism.	No clear description of analytical procedures. Theory-based interpretation of data. Results are categorised by aspects of reciprocal determinism.	Home and school FV availability, access to unhealthy food in school, sensory attributes (taste, appeal, appearance, smell, mouth feel), methods of preparation, preferences/liking, outcome expectancies, acceptance of national recommendations, food categorisation, preparation skills.

**Bauer et al. 2004 **[57]	US	Healthy nutrition (and physical activity)	School-based (2 schools): 26 schoolchildren and 23 faculty and staff members.	A: mixed. B: no info. C: 7 & 8. D (school level): mixed composition. E: mixed composition (80% White, 20% either Asian- or African- Americans).	FG discussions: 7 grade- and gender-homogeneous schoolchild groups, 3 faculty and staff member groups and 10 individual interviews with key informants (e.g. school nurse, cafeteria manager, administratives).	Ecological models by Bronfenbrenner (1979), Stokols (1996), Story, Neumark-Stzainer & French (2002).	Grounded theory: 1st step: systematic coding of themes, 2nd step: identification of 3 mechanisms of influences on eating within the school environment based on data-developed concepts and theoretical framework.	School FV availability (quantity, variety, quality), School availability of unhealthy competitive food choices.

**Booth et al. 2008 **[49]	Australia	Healthy food (Perceived causes of overweight and obesity)	School-based (3 secondary schools): 58 schoolchildren.	A: mixed, B: 12-17. C: 7-11. D (area level): areas selected to reflect a wide range of SEP differences. E: No info.	9 gender- and school year-homogeneous FGs (year 7+8, year 9+10, year 11).	No info.	No clear description of analytical procedures. Coding of themes.	School FV availability (price, quality, presentation).

**Campbell 2009 **[30]	US	Dietary choices	School-based (1 school): 12 schoolchildren.	A: mixed. B: 14-16. C: 9 & 10. D: pre-dominantly low-income families. E: mixed (Hispanic, African-American, Eurasian and combination of these).	FG discussions: one group interviewed twice, during lunch and immediately after.	Developmental psychology by Piaget and Erikson.	Content analysis, but no clear description of analytical procedures.	Home and school FV availability, parental influence, availability, liking, methods of preparation, knowledge, food categorisation.

**Cullen et al. 1998 **[25]	US	FV con-sumption	Community-based: 99 urban boy scouts and 39 parents.	A: boys. B: 10-14. C: elementary school. D: no info. E: mainly African-American (88%).	13 FGs with boy scouts and 7 FGs with parents.	Social cognitive theory concept of reciprocal determinism.	No clear description of analytical procedures. Transcripts were coded and quantified.	Preferences, outcome expectancies, sensory attributes (taste, mouth feel), snack food purchases, price, parental-, peer-, and media influence, preparation skills, home accessibility, school availability.

**Cullen et al. 2000 **[48]	US	FJV intake	School-based (6 schools): 180 schoolchildren and 40 parents.	A: no info. B: 9-12. C: 4-6. D: mixed. E: mixed: 3 Afro-American schools, 1 Euro-American school, 2 Mexican-American schools.	School year and ethnically homogeneous FG discussions: 6 African-American schoolchild FGs, 6 Euro-American schoolchild FGs, 5 Mexican-American schoolchild FGs, and 8 Parent FGs.	Social cognitive theory: reciprocal determinism.	Data-based analysis. Systematic coding of transcripts and comparisons of results by ethnicity. Data-based variable names assigned to text passages.	Home availability/accessibility (variety), peer-, parental-, and media influence, sensory attributes (taste), food categorisation.

**Cullen et al. 2007 **[26]	US	School food	School-based (6 schools): schoolchildren, school staff and district school food administrators (no. of participants not provided).	A: no info. B: 11-14. C: middle school. D (school-level): at least 50% of schoolchild population received free or reduced price meals. E (school-level): at least 50% of schoolchild population was African- American and Hispanic.	11 FGs with schoolchildren/school staff. Interviews with 7 district school food administrators.	No info.	No clear description of analytical procedures.	School V availability (variety, freshness).

**Evans et al. 2006 **[40]	US	Healthful eating	48 adolescents from two middle schools and one recreation & parks centre.	A: mixed. B: 10-14. C: 6 & 7. D: low income. E: Mainly Black (81%).	3 male and 2 female FGs.	Social cognitive theory	Systematic analysis based on pre-specified coding scheme (categorisation of data according to gender, location, and motivational theme) and standardised procedures.	Nutritional knowledge/outcome expectations (misconceptions), home availability (access to competitive food choices), peer pressure/symbolic value of food, school availability (appearance, appeal, freshness), FV availability at restaurants. Gender differences.

**Fitzgerald et al. 2009 **[41]	Australia	Eating behaviour (and physical activity)	School-based (1 school): 37 schoolchildren.	A: mixed. B: no info. C: kindergarten & year 1-6. D: low SEP community. E: no info.	3 FGs: kindergarten + year 1-2, year 3-4, year 5-6.	The socio-ecological approach is cited in the introduction.	Open coding/thematic analysis of transcripts.	Outcome expectancies, adult-, peer- and media influence, symbolic value of food, sensory attributes (taste), convenience, access to unhealthy food in local area, time limitations.

**Gellar et al. 2007 **[31]	US	Healthy eating	140 youth from diabetes camp.	A: mixed. B: 7-16 (mean age: 11.8). C: no info. D: mixed. E: mixed (71% white, 18% Black, 6% Hispanic).	12 female and 6 male FGs (almost similar age).	No info.	Content analysis: Systematic coding of transcripts using a pre-specified coding system.	Preferences, sensory attributes (taste), knowledge, outcome expectancies, school FV availability (appeal, methods of preparation/form, competitive unhealthy food choices), convenience, home availability of unhealthy food, peer- and parental influence.

**Goh et al. 2009 **[53]	US	Healthy eating (and physical activity)	School-based (2 schools): 119 schoolchildren, 63 parents, and 28 key stakeholders.	A: mixed. B: mean age: 12. C: 7 & 8. D: no info. E: mixed (58% Latino).	6 male and 8 female schoolchild FGs, 8 parent FGs, interviews with 28 key stakeholders.	No info.	Systematic content analysis.	School availability (accessibility, appearance, methods of preparation, visibility, braces-friendly FV, unhealthy food), knowledge, parental influence.

**Hill et al. 1998 **[32]	New Zealand	FV con-sumption	Community-based: 20 teenagers and their parent.	A: mixed. B: 13-16. C: no info. D: mixed. E: Pakeha (European ancestry).	20 interviews: Separate interviews with teenager and parent responsible for food preparation.	No info.	Cross-household analysis. The analysis focus on interaction between teenager and parent-shopper in each household. No clear description of analytical procedures.	Situational norms, convenience, FV preparation skills, FV availability at home, school, and in local area (appeal, quality, parental facilitation, price, variety), peer-, parental- and media influence, preferences, outcome expectancies, knowledge. Age and gender differences.

**Husby et al. 2008 **[51]	Denmark	Meals & snack consumption	Children with a healthy diet (N = 9) and a less healthy diet (N = 8) were recruited through a dietary survey among their parents.	A: mixed. B: 10-11. C: no info. D: mixed. E: no info.	17 photo-elicited, semi-structured individual interviews.	Meals are examined as social events. Meals involve the establishment and re-establishment of the family unit.	Template analysis (pre-specified themes) and comparative analysis.	Peer influence (food swapping), snack, outcome expectancies, FV preparation skills, parental facilitation, food rules. Gender differences.

**Keim et al. 2001 **[33]	US	FV intake	Community-based: 27 Caucasian and 30 Mexican-American healthy, low income children from public school, migrant worker summer schools and community centres.	A: mixed. B: 8-11. C: 3. D: low income. E: Caucasian- and Mexican-American.	FG discussions: 4 FGs of Caucasian children and 6 FGs of Mexican-American children.	Social cognitive theory	Transcripts analysed and coded within the context of Social cognitive theory.	Parental facilitation, FV preparation skills, FV shopping, price, home availability/accessibility (visibility, convenience, variety, unhealthy food), parental- and peer influence, preferences, sensory attributes (taste, mouth feel, appearance, quality, freshness, methods of preparation, familiarity), outcome expectancies, knowledge. Ethnic differences.

**Khunti et al. 2008 **[50]	UK	Healthy lifestyle	School-based: Pupils (no. not provided but can be estimated to maximum 144) and school staff.	A: mixed. B: 11-15. C: 7-10. D: schools located in a very deprived area. E: mixed: In the overall sample 77% of the pupils were of South Asian origin.	Action research approach. Baseline: 18 schoolchild- and 5 staff FGs. Follow-up: 8 schoolchild- and 5 staff FGs. Observational visits at all schools.	No info.	Open coding (in line with the 1st analytical step of grounded theory) of data. A process of progressive focussing is used to develop a thematic framework.	Peer influence (image), cost & risk of wasting money, hunger satisfaction.

**Kim et al. 2007 **[42]	US	Dietary practices/FV intake (and physical activity)	Community-based: Low-income Hmong American parents (N = 44) and youth (N = 40). Key informants (N = 5) in Hmong communities.	A: mixed. B: 11-14. C: no info. D: low-income. E: Hmong Americans.	8 FGs with adults and youths and 5 individual interviews with key informants.	No info.	No clear description of analytical procedures. The transcripts were coded and organised.	Outcome expectancies, knowledge, preferences, parental influence, sensory attributes (smell, freshness), time/occasions for eating FV, school availability.

**Kirby et al. 1995 **[34]	US	FV intake	School-based (6 schools from 3 regions): 398 schoolchildren, 108 parents, 43 teachers, 29 school food service workers.	A: no info. B: no info. C: 4-5. D (region level): mixed. E: mixed. 2 schools of predominantly white. Caucasian ethnic composition (1 high and 1 middle SEP) and 4 schools of non-white (African-, Asian-, Hispanic American, other or multi-ethnic) composition (2 Low and 2 very low SEP).	FG discussions: 15 schoolchild- (school year homogeneous), 11 parent-, 6 teacher- and 6 Food service worker-FGs.	Social learning theory: reciprocal determinism.	Systematic, theory-guided coding of transcripts. The assigned variable names were developed based on the discussion guide and theoretical framework.	Home availability/accessibility (variety, parental facilitation), preparation skills, price, preferences (variety liked), sensory attributes (taste, mouth feel), food categorisation, knowledge, convenience, methods of preparation, outcome expectancies, time/occasions for eating FV (restaurants), peer influence, availability in local area/restaurants. SEP differences.

**Kubik et al. 2005 **[35]	US	Dietary practice (and physical activity)	School-based (7 Alternative High Schools): 70 schoolchildren.	A: mixed. B: no info. C: 9-12. D: (school-level): mixed composition: 46% of schoolchildren qualified for free reduced lunch program. E: mixed composition: 36% of schoolchildren were of non-Caucasian origin (American-Indian, African-American, Hispanic, Asian).	7 schoolchild FGs.	Ecological theory and social learning theory.	Systematic 3-step analytical process as described by Miles & Huberman (1994).	Convenience, home and school FV availability/accessibility, access to unhealthy competitive food in school and local area, price, quality, preferences, cooking skills.

**Lauten-schlager & Smith 2007 **[36]	US	Dietary behaviour (values, beliefs and gardening & cooking behaviours)	Community-based: 40 inner-city youth. Two subgroups: involved in Youth Farm Garden Program (N = 26) and not involved (N = 14).	A: mixed. B: 9-15. C: no info. D: no info. E: mixed: white (15%), African-American (30%), Hispanic (17%), Asian (27%), Somali (7%), other or multiracial (14%).	6 FGs: 3 with garden program participants and 3 with youth not involved in garden program.	Theory of planned behaviour	Application of systematic, content analysis procedures by Miles & Huberman (1994).	Sensory attributes (flavour/taste, mouth feel/texture, appearance) convenience, preferences, method of preparation, outcome expectancies, knowledge, availability in the neighbourhood (seasonality, quality, quantity, supply), parental-, peer- and media influence.

**Libman 2007 **[43]	US	Food conscious-ness and V eating habits	Community-based: 10 schoolchildren from a children garden program, four mothers and one father.	A: mixed. B: 10-14. C: no info. D: no info. E: African-American, Puerto Rican, Dominican, and Guyanese.	Schoolchildren: 1 FG, 10 semi-structured seated interviews, 6 walking interviews (youth-led garden tours). 5 parent telephone interviews. Observations of program and material.	Developmental psychology by Lev Vygotsky.	Systematic coding for themes relevant to research questions (Miles & Huberman 1994).	Sensory attribute (taste, methods of preparation), cooking skills, food consciousness/knowledge, home FV availability (appearance, freshness, safety of organic FV).

**McKinley et al. 2005 **[44]	England and Northern Ireland	Healthy eating	School-based (11 schools):106 schoolchildren.	A: Mixed, B: 11-12. C: 1st year of post-primary school. D (school level): mixed SEP backgrounds. E: mixed ethnic backgrounds: White Europeans (76%), Asian (18%), Afro-Caribbean (6%).	11 FGs (2 discussion sessions per group). 4 of the FGs were gender-homogeneous as they were conducted at single-sex school.	No info.	Systematic coding of transcripts using the cut-and-paste technique described by Stewart & Shamdasini (1990).	Food categorisation, school availability (appearance, quality), sensory attributes (texture, mouth feel), convenience & time costs, cost & taste guarantee, cost & filling power, rebellion. Gender differences.

**Molaison et al. 2005 **[37]	US	FV intake	Community-based: 42 southern, low-income black American adolescents recruited from National Youth Sport Program.	A: Mixed. B: 10-13. C: no info. D: low income. E: Black Americans.	6 gender- and age-homogeneous FGs.	Social cognitive theory	Theory guided the analysis. Transcripts were coded by content analysis methods and codes/themes were assigned to the theoretical framework.	Sensory attribute (taste, method of preparation, form (canned vs. fresh)), allergies, preferences, variety (vegetable boredom), outcome expectancies, food preparation skills, home and neighbourhood availability, appropriate settings for FV, family- and peer influence, self-efficacy. Gender differences.

**Monge-Rojas et al. 2005 **[28]	Costa Rica	Healthful eating	School-based (3 schools): 108 schoolchildren.	A: mixed. B: 12-18. C: 7-11. D (school-level): mixed (2 public high schools and 1 private high school). E: Costa Rican.	12 gender- and age- homogeneous FGs (3 sessions per group).	Conceptual model for adolescent eating behaviours based on Social cognitive theory and ecological perspective proposed by Story et al. (2002).	The transcripts were reviewed systematically for emerging themes. Themes were identified according to the theoretical framework.	Knowledge, school availability of FV and unhealthy food, home availability, parental facilitation, peer influence/norms (gender roles, symbolic value of food), cost & satiety value, sensory attributes (taste, methods of preparation), convenience & time considerations, outcome expectations, parental- and media influence. Gender differences.

**Neumark-Stzainer et al. 1999 **[45]	US	Food choices and eating behaviours	School-based (2 schools): 141 schoolchildren.	A: mixed. B: 12-14 (mean age: 12.6) & 15-19 (mean age: 16.0). C: 7 & 10. D: no info. E: mixed composition: white (40%), Asian-American (25%), African-American (21%), multiracial (7%), Hispanic (6%), Native American (1%).	21 age- and gender-homogeneous FGs.	Social cognitive theory is included in the discussion.	Systematic analytical approach using the constant comparative method of grounded theory.	Sensory attributes (taste, appeal, appearance, methods of preparation), convenience & time considerations, hunger satisfaction & costs, availability at home, school and fast food restaurants (visibility, accessibility). Gender differences.

**Nicklas et al. 1997 **[27]	US	FV intake	School-based (4 schools): 55 high school schoolchildren.	A: mixed. B: no info. C: 9. D: no info. E: mixed. Participants drawn from a student cohort of mainly Caucasian background (79%). The rest are of Hispanic, African-American, Asian or Native American origin.	4 FGs (white male, white female, black male, black female) - unclear if FGs mix schoolchildren from different schools.	The intervention is based on the PRECEDE model of health education (6 levels of behaviour change).	No clear description of analytical procedures.	Outcome expectancies, sensory attributes (taste), inconsistency in taste, home & school FV availability (visibility, variety, presentation/appearance), cost, access to competitive unhealthy food in school and local area.

**O'Dea 2003 **[52]	Australia	Healthful food (and physical activity)	School-based (34 schools): 213 schoolchildren and 38 school principals.	A: mixed. B: 7-17. C: 2-11. D & E: a representative mix of SEP and ethnicity.	38 FGs.	Theory of planned behaviour and social learning theory.	Content analysis (Miles & Huberman), systematic approach.	Outcome expectancies, food categorisation, knowledge, home availability (unhealthy competitive food choices), convenience & time costs.

**Ross 1995 **[46]	Scotland	Food choices and preferences	School-based (one school): 46 schoolchildren.	A: mixed. B: 10-12 (mean age: 11). C: primary 7 year. D (area): School situated in catchment area encompassing all SEP groups. E: schoolchildren were predominantly white (only a few from ethnic minority backgrounds).	FG discussions: 2 male FGs, 3 female FGs and 2 mixed-gender FGs. Planned observations during lunch time were not feasible because of the fact that lunch occurred in several sites simultaneously and only one researcher being involved in the project.	No info.	Grounded theory approach	Sensory attributes (taste, texture), peer norms/influence (food swapping, socially acceptable food), affordability.

**Steven-son et al. 2007 **[47]	Ireland	Healthy eating	School-based (no info. on number of schools): 73 adolescents.	A: mixed. B: 12-15. C: second level schools. D: mixed. E: no info.	12 age- and gender-homogeneous FGs.	Socio-ecological approach.	Systematic coding of transcripts and deviant case analysis.	Sensory attributes (taste), parental influence.

**Walker et al. 1973 **[16]	US	FV intake	Schoolchildren and parents (primarily mothers). No. not provided, but can be estimated to maximum 220 participants.	A: mixed. B: 9-12 & 13-17. C: elementary and high school. D: middle and low income families. E: no info.	FG discussions (school year-gender-SEP homogeneous): 8 elementary schoolchild FGs (2 boy- & 2 girl-low income FGs and 2 boy- & 2 girl-middle income FGs), 8 high school student FGs, and 6 parent groups (3 low and 3 middle income FGs).	No information. Study conducted by social psychologists.	No clear description of analytical procedures.	Availability/exposure to FV at home and in local area (variety), price, parental style/attitude, preferences, sensory attributes (appearance, colour, texture, taste, odour, form, method of preparation), food prejudices.

**Wind et al. 2005 **[38]	The Netherlands and Belgium-Flanders	FV intake	School-based: 3 schools from the Netherlands, 60 schoolchildren. 32 schoolchildren from Belgium, no. of schools not provided.	A: mixed. B: 10-11. C: 5-6. D: no info. E: **Netherlands**: In two of the schools almost all children were from ethnic minority groups, in one school all except one child had both parents born in the Netherlands. **Belgium-Flanders**: 4 children had parents born in a foreign country.	FG discussion: **Netherlands**: 2 boy FGs, 1 girl FG, 5 mixed gender FGs. **Belgium-Flanders**: 1 boy FG, 1 girl FG, 2 mixed gender FGs.	Health belief model, theory of planned behaviour, social ecological models.	No clear description of analytical procedures. Determinants are analysed separately for fruit and vegetables. Determinants classified as personal, home- or school environmental factors.	Outcome expectancies, food categorisation, sensory attributes (taste, appearance, texture), preferences, knowledge/awareness, preparation skills, situational/social norms (time/settings for eating FV), convenience, home and school availability/accessibility (visibility, family rules, parental facilitation), unhealthy food shopping, peer-, parental and teacher influence. Ethnic and international differences.

**Zeinstra et al. 2007 **[39]	The Netherlands	FV preferences	School-based (1 school): Schoolchildren representing 3 different stages of cognitive development.	A: mixed. B: 4-5 (group A), 7-8 (group B) and 11-12 (group C). C: 1st, 4th and last school year of primary school. D: no info. E: no info.	4 + 4 duo interviews with group A and B and 4 FGs with group C.	Cognitive theory (Piaget).	Transcripts were coded systematically using a coding framework based on research aims, the interview guide and previous findings in the literature.	Preferences, sensory attributes (taste, texture, appearance, methods of preparation, familiarity, food categorisation), outcome expectancies, appropriate time and occasions for eating FV. Age differences.

**Abbreviations: **FV = fruit and vegetables; FG = focus group; Info: information; FJV = Fruit, juice and vegetables; No. = number: Ref. ID = ID number of study in the reference list; SEP = socioeconomic position; US = the United States of America; V = vegetables; vs. = versus; UK = the United Kingdom.

### II. Evaluation of papers: overall quality assessment

We evaluated 13 studies to be of high methodological quality and 12 and six studies to be of medium and low methodological quality respectively (based on agreement between at least two of the reviewers). The high quality studies were characterised by a transparent and systematic research process (explicit aims and a clear presentation of preconceptions, sampling, data collection, data analysis, findings, and validation of findings) and included a thorough discussion of transferability of findings, study limitations and contribution of the study to the previous knowledge base. The common characteristics of the studies of low methodological quality (table 2) were insufficient description of aims, preconceptions, data collection and analysis and lack of discussion of study limitations, transferability of findings and the contribution of the study. However, for two of these studies a thorough description of the qualitative study was not the main aim of the paper [26,27].

**Table 2:** Quality assessment scheme (Y indicates 'yes, information is provided'; NA indicates that the criterion is not applicable or relevant for the study)

Study listed alphabetically by last name of first author (B-Kh)	Baranowski et al. (1993)	Bauer et al. (2004)	Booth et al. (2008)	Campbell (2009)	Cullen et al. (1998)	Cullen et al. (2000)	Cullen et al. (2007)	Evans et al. (2006)	Fitzgerald et al. (2009)	Gellar et al. (2007)	Goh et al. (2009)	Hill et al. (1998)	Husby et al. (2008)	Keim et al. (2001)	Khunti et al. (2008)	
**Aims**																

Aims and research questions are explicitly stated	Y	Y		Y	Y	Y		Y	Y	Y	Y		Y	Y	Y	

Qualitative approach appropriate to answer research questions	Y	Y	Y	Y	Y	Y	Y	Y	Y	Y	Y	Y	Y	Y	Y	

**Preconceptions**																

Explicit theoretical framework or literature review and/or pre-study beliefs	Y	Y	Y	Y	Y	Y		Y	Y	Y	Y	Y	Y	Y		

Information on how theory is used (NA if no theoretical framework)	Y	Y			Y	Y		Y		NA	NA	NA		Y		

**Sampling procedure**																

Explicit sampling strategy of field sites and/or of children		Y	Y	Y		Y	Y	Y	Y			Y	Y	Y	Y	

Recruitment strategy: how?	Y	Y	Y				Y	Y	Y	Y	Y	Y	Y	Y	Y	

Recruitment strategy: by whom?		Y	Y			Y		Y	Y	Y	Y	Y		Y	Y	

Explicit justification of sampling strategy	Y	Y	Y					Y	Y		Y	Y	Y			

Sampling strategy reflects the study purpose		Y	NA	Y		Y	Y	Y	Y	Y	Y	Y	Y	Y	Y	

Sample size provided or can be estimated	Y	Y	Y	Y	Y	Y		Y	Y	Y	Y	Y	Y	Y	Y	

Non-participation described/response rate (NA if voluntary sample)				NA	Y	Y		Y		Y			Y	Y		

Sampling/data collection continued until point of data saturation											Y			Y		

**Ethical concerns: explicit statement about.....**.																

Informed consent (parental or child)	Y	Y	Y	Y	Y	Y	Y	Y	Y	Y	Y		Y	Y		

Anonymity and confidentiality			Y	Y											Y	

Ethical approval/review		Y	Y	Y		Y	Y	Y	Y	Y	Y			Y	Y	

**Sample characteristics: Explicit and sufficient description of.....**.																

Gender of child participants		Y	Y	Y	Y			Y	Y	Y	Y	Y	Y	Y	Y	

Age of child participants or school year	Y	Y	Y	Y	Y	Y	Y	Y	Y	Y	Y	Y	Y	Y	Y	

Socioeconomic background of child participants	Y			Y		Y		Y		Y		Y	Y	Y		

Ethnic background of child participants	Y	Y		Y	Y	Y		Y		Y	Y	Y		Y	Y	

Other study-specific characteristics of child participants	NA	NA	Y	Y	NA	Y	NA	Y	NA	Y	Y	Y	Y	NA		

**Data collection**																

Data collection method (e.g. focus groups, observations) stated	Y	Y	Y	Y	Y	Y	Y	Y	Y	Y	Y	Y	Y	Y	Y	

Explicit rationale for data collection method	Y	Y		Y		Y		Y	Y	Y			Y			

Data collection methods adequate to answer research questions	Y	Y	Y	Y	Y	Y	Y	Y	Y	Y	Y	Y	Y	Y	Y	

Number of focus groups, interviews, observations provided	Y	Y			Y	Y	Y	Y	Y	Y	Y		Y	Y	Y	

Size of focus groups described or average can be estimated	Y	Y	Y		Y	Y		Y	Y	Y	Y	NA	NA	Y	Y	

Composition of child focus groups/interviews described	Y	Y				Y		Y	Y	Y	Y		Y	Y		

Explicit rationale for focus group/interview composition		Y				Y			Y	Y	Y		NA	Y		

Interview setting described	Y		Y	Y	Y	Y		Y		Y	Y		Y	Y		

Interviewer described (who?, how many?)	Y	Y	Y	Y	Y	Y		Y	Y	Y	Y		Y		Y	

Duration of interviews, focus groups, observations described		Y	Y	Y	Y	Y		Y		Y	Y	Y	Y	Y		

**Interview guide **																

Interview guide used	Y	Y	Y	Y	Y	Y	Y	Y	Y	Y	Y		Y	Y	Y	

If yes: Partly described (key questions)? Y, fully described? YY	Y	Y	Y	YY	Y	Y	Y	YY		YY	YY		Y	YY		

**Analysis**																

***Reliability/consistency***																

Explicit information of audiotaping of interviews		Y	Y	Y	Y	Y		Y	Y	Y	Y		Y	Y		

Explicit information of transcription of interviews Y, verbatim: YY		YY	YY	Y	Y	Y		YY	Y	YY	Y	Y	YY	Y		

***Communicative validity***																

Analyst described (who?, how many?)		Y	Y					Y	Y	Y	Y			Y		

Clear description of analytical method?		Y				Y		Y	Y	Y	Y		Y	Y	Y	

Explicit analytical approach (data-based or theory-based)		Y				Y		Y	Y	Y	Y		Y	Y	Y	

Analytical procedures appropriate to the research questions		Y				Y		Y	Y	Y	Y		Y	Y	Y	

Explicit rationale for choice of analytical procedures												Y				

Sampling strategy/ child focus group composition is used in analysis			Y		NA	Y		Y				Y	Y	Y	NA	

**Findings/ presentation of findings**																

Clear presentation of findings		Y	Y	Y	Y	Y		Y	Y	Y	Y	Y	Y	Y	Y	

Authors' voices can always be distinguished from informants' voices	Y	Y	Y	Y	Y	Y	Y	Y	Y	Y	Y	Y		Y	Y	

Sufficient inclusion of quotes to support findings		Y	Y					Y	Y	Y	Y	Y	Y	Y		

Clear description of selection and edition of quotes													Y			

Different child participants' views can be distinguished		Y							Y			Y	Y			

The stated conclusion is supported by the findings	Y	Y	Y		Y	Y		Y	Y	Y	Y	Y	Y	Y	Y	

Relevance: Findings/conclusions illuminate the research questions	Y	Y	NA		Y	Y		Y	Y	Y	Y	Y	Y	Y	Y	

**Internal validity**																

Description of validity and pilot-testing of applied instruments/guides	Y	Y	Y		Y				Y					Y		

***Explicit strategies for validating presented findings***																

Researcher/analyst triangulation			Y			Y		Y	Y	Y	Y			Y		

Method triangulation		Y		Y	Y		Y		Y		Y		Y		Y	

Source triangulation	Y	Y			Y	Y	Y				Y	Y			Y	

Theory triangulation																

Peer debriefing/audit trail	Y		Y							Y	Y					

Member checks/respondent validation																

Attention to negative or deviant cases																

**External validity**																

Discussion of transferability (applicability of findings in other contexts)		Y				Y	Y	Y	Y	Y	Y			Y	Y	

Explicit reflections on selection bias/non-response of children		Y														

**Discussion**																

Adequate attention to previous knowledge and what the study adds	Y	Y		Y	Y	Y		Y			Y	Y	Y	Y	Y	

Findings provide new insight on potential determinants of fruit and vegetables	Y	Y		Y	Y	Y	Y	Y	Y	Y	Y	Y	Y	Y	Y	

Discussion of limitations of qualitative study	Y	Y				Y		Y	Y		Y			Y	Y	

**Pragmatic validity**																

Discussion of implications for research and practice	Y	Y		Y	Y	Y	Y	Y	Y	Y	Y	Y	Y	Y		

**Quantitative quality score: Total number of quality requirements met **	**32**	**49**	**32**	**32**	**33**	**44**	**19**	**49**	**42**	**46**	**48**	**31**	**43**	**48**	**32**	

**Qualitative quality score evaluated by reviewers** **(H: high, M: medium, L: low)**	**M**	**H**	**L**	**L**	**M**	**H**	**L**	**H**	**M**	**M**	**H**	**M**	**M**	**H**	**M**	

**Study listed alphabetically by last name of first author (Ki-Z)**	**Kim et al. (2007)**	**Kirby et al. (1995) **	**Kubik et al. (2005)**	**Lautenschlager & Smith (2007) **	**Libman (2009)**	**McKinley et al. (2005)**	**Molaison et al. (2005)**	**Monge-Rojas (2005)**	**Neumark-Stzainer et al. (1999) **	**Nicklas et al. (1997)**	**O'Dea (2003)**	**Ross (1995)**	**Stevenson et al. (2007) **	**Walker et al. (1973)**	**Wind et al. (2005)**	**Zeinstra et al. (2007)**

**Aims**																

Aims and research questions are explicitly stated	Y	Y	Y	Y	Y	Y	Y	Y	Y	Y	Y	Y	Y	Y	Y	Y

Qualitative approach appropriate to answer research questions	Y	Y	Y	Y	Y	Y	Y	Y	Y	Y	Y	Y	Y	Y	Y	Y

**Preconceptions**																

Explicit theoretical framework or literature review and/or pre-study beliefs		Y	Y	Y	Y	Y	Y	Y	Y	Y	Y	Y	Y		Y	Y

Information on how theory is used (NA if no theoretical framework)		Y	Y	Y		NA	Y	Y	Y	Y	Y	NA			Y	Y

**Sampling procedure**																

Explicit sampling strategy of field sites and/or of children	Y	Y	Y	Y		Y	Y	Y	Y		Y	Y	Y	Y	Y	

Recruitment strategy: how?	Y	Y	Y	Y		Y	Y	Y	Y	Y	Y	Y	Y		Y	Y

Recruitment strategy: by whom?	Y		Y	Y				Y		Y	Y	Y	Y			

Explicit justification of sampling strategy		Y	Y	Y		Y	Y	Y	Y		Y	Y	Y	Y	Y	Y

Sampling strategy reflects the study purpose	Y	Y	Y	Y	Y	Y	Y	Y	Y		Y	Y	Y	Y	Y	Y

Sample size provided or can be estimated	Y	Y	Y	Y	Y	Y	Y	Y	Y	Y	Y	Y	Y		Y	Y

Non-participation described/response rate (NA if voluntary sample)	NA	Y	Y		Y			Y	Y		Y	Y		Y		

Sampling/data collection continued until point of data saturation				Y												

**Ethical concerns: explicit statement about.....**.																

Informed consent (parental or child)		Y	Y	Y		Y	Y	Y	Y		Y		Y		Y	Y

Anonymity and confidentiality						Y	Y						Y		Y	Y

Ethical approval/review	Y	Y	Y	Y		Y	Y	Y							Y	

**Sample characteristics: Explicit and sufficient description of.....**.																

Gender of child participants	Y		Y	Y	Y	Y	Y	Y	Y	Y	Y	Y	Y	Y	Y	Y

Age of child participants or school year	Y	Y	Y	Y	Y	Y	Y	Y	Y	Y	Y	Y	Y	Y	Y	Y

Socioeconomic background of child participants	Y						Y				Y		Y	Y		

Ethnic background of child participants	Y		Y	Y	Y	Y	Y	Y	Y	Y	Y	Y			Y	

Other study-specific characteristics of child participants	NA	Y	Y	Y		Y	NA	Y	NA	NA	NA	NA	Y	Y	Y	NA

**Data collection**																

Data collection method (e.g. focus groups, observations) stated	Y	Y	Y	Y	Y	Y	Y	Y	Y	Y	Y	Y	Y	Y	Y	Y

Explicit rationale for data collection method		Y	Y		Y	Y		Y	Y	Y		Y	Y	Y	Y	Y

Data collection methods adequate to answer research questions	Y	Y	Y	Y	Y	Y	Y	Y	Y	Y	Y	Y	Y	Y	Y	Y

Number of focus groups, interviews, observations provided	Y	Y	Y	Y	Y	Y	Y	Y	Y	Y	Y	Y	Y	Y	Y	Y

Size of focus groups described or average can be estimated		Y	Y	Y		Y	Y	Y	Y	Y		Y	Y	Y	Y	Y

Composition of child focus groups/interviews described		Y	Y	Y			Y	Y	Y	Y		Y	Y	Y	Y	Y

Explicit rationale for focus group/interview composition				Y				Y					Y			Y

Interview setting described		Y	Y		Y	Y	Y	Y	Y			Y	Y			Y

Interviewer described (who?, how many?)	Y	Y	Y	Y		Y	Y	Y	Y	Y		Y	Y	Y	Y	Y

Duration of interviews, focus groups, observations described	Y		Y	Y	Y	Y		Y	Y		Y	Y	Y	Y	Y	Y

**Interview guide **																

Interview guide used	Y	Y	Y	Y	Y	Y	Y	Y	Y	Y	Y	Y	Y	Y	Y	Y

If yes: Partly described (key questions)? Y, fully described? YY		YY	Y	YY	Y	YY	YY	Y	Y	Y	Y	YY	Y	Y	Y	YY

**Analysis**																

***Reliability/consistency***																

Explicit information of audiotaping of interviews	Y	Y	Y	Y	Y	Y	Y	Y	Y	Y	Y	Y	Y	Y	Y	Y

Explicit information of transcription of interviews Y, verbatim: YY	Y	YY	YY	YY	Y	YY	YY	YY	YY		YY	YY	Y		Y	Y

***Communicative validity***																

Analyst described (who?, how many?)		Y	Y	Y		Y	Y	Y	Y			Y		Y	Y	Y

Clear description of analytical method?		Y	Y	Y	Y	Y	Y	Y	Y		Y	Y	Y			Y

Explicit analytical approach (data-based or theory-based)		Y	Y	Y	Y	Y	Y	Y	Y		Y	Y	Y		Y	Y

Analytical procedures appropriate to the research questions		Y	Y	Y	Y	Y	Y	Y	Y		Y	Y	Y		Y	Y

Explicit rationale for choice of analytical procedures		Y	Y			Y	Y	Y	Y				Y	Y	Y	Y

Sampling strategy/ child focus group composition is used in analysis		Y	Y	Y		Y	Y	Y	Y	Y	Y		Y	Y	Y	Y

**Findings/ presentation of findings**																

Clear presentation of findings	Y	Y	Y	Y	Y	Y	Y	Y	Y	Y	Y	Y	Y	Y	Y	Y

Authors' voices can always be distinguished from informants' voices	Y	Y	Y	Y	Y	Y	Y	Y	Y	Y	Y	Y	Y	Y	Y	Y

Sufficient inclusion of quotes to support findings			Y	Y	Y	Y	Y	Y	Y	Y	Y	Y	Y		Y	

Clear description of selection and edition of quotes				Y				Y	Y							

Different child participants' views can be distinguished			Y	Y				Y	Y		Y	Y	Y			

The stated conclusion is supported by the findings	Y	Y	Y	Y	Y	Y	Y	Y	Y	Y	Y	Y	Y	Y	Y	Y

Relevance: Findings/conclusions illuminate the research questions	Y	Y	Y	Y	Y	Y	Y	Y	Y	Y	Y	Y	Y	Y	Y	Y

**Internal validity**																

Description of validity and pilot-testing of applied instruments/guides		Y	Y		Y	Y	Y	Y	Y			Y	Y		Y	Y

***Explicit strategies for validating presented findings***																

Researcher/analyst triangulation			Y	Y		Y	Y	Y	Y						Y	Y

Method triangulation	Y				Y					Y		Y				Y

Source triangulation	Y	Y			Y						Y			Y		

Theory triangulation																

Peer debriefing/audit trail					Y			Y	Y					Y	Y	Y

Member checks/respondent validation																

Attention to negative or deviant cases													Y			

**External validity**																

Discussion of transferability (applicability of findings in other contexts)	Y	Y	Y		Y		Y	Y		Y		Y			Y	Y

Explicit reflections on selection bias/non-response of children	Y				Y			Y	Y							

**Discussion**																

Adequate attention to previous knowledge and what the study adds			Y	Y		Y	Y	Y	Y		Y	Y	Y		Y	Y

Findings provide new insight on potential determinants of fruit and vegetables	Y	Y	Y	Y	Y	Y	Y	Y	Y	Y	Y	Y	Y	Y	Y	Y

Discussion of limitations of qualitative study	Y	Y	Y		Y	Y	Y	Y	Y	Y		Y		Y	Y	Y

**Pragmatic validity**																

Discussion of implications for research and practice	Y	Y	Y	Y	Y	Y	Y	Y	Y	Y	Y	Y	Y	Y	Y	Y

**Quantitative quality score: Total number of quality requirements met **	**33**	**44**	**50**	**47**	**36**	**47**	**48**	**54**	**50**	**32**	**40**	**47**	**45**	**33**	**46**	**47**

**Qualitative quality score evaluated by reviewers** **(H: high, M: medium, L: low)**	**L**	**H**	**H**	**H**	**M**	**H**	**H**	**H**	**H**	**L**	**L**	**M**	**M**	**M**	**M**	**H**

In addition to the overall assessment of the methodological quality, we also counted the number of quality criteria met out of 63 possible. The studies in the highest third met more than 46 quality criteria (11 assessed as high and 1 medium quality), studies in the medium third met 36-46 criteria (2 assessed as high, 6 medium quality and 1 low quality), and studies in the lowest third (5 assessed as medium and 5 low quality) met less than 34 criteria. Maximum number of quality requirements (54 out of 63) were met in the study by Monge-Rojas et al. (2005) and minimum (19 out of 63) in the study by Cullen et al. (2007) [26,28].

### III. Synthesis of findings from the reviewed studies

We identified sixteen main themes 1) Preferences/liking and variety, 2) Sensory and physical attributes of fruit and vegetables, 3) Satiety value/hunger satisfaction/filling power, 4) Outcome expectations/expectancies, 5) Knowledge, 6) Food categorisation: perceptions of fruit and vegetables, 7) Fruit and vegetable preparation skills (behavioural capability), 8) Convenience and time costs, 9) Price and affordability, 10) Situational norms: Appropriateness of time, settings/locations and occasions for eating fruit and vegetables, 11) Availability and exposure to fruit and vegetables at home, 12) Availability and exposure to fruit and vegetables in school, 13) Availability and exposure to fruit and vegetables in neighbourhood/local area, and 14) Parental influence, 15) Peer influence, and 16) TV/media influence. The content of each main theme is described below. Within each theme we have highlighted the country origin of the finding if there were inconsistent findings across countries, or if the theme was only discussed in one or a few country settings. No mentioning of study setting in relation to a theme indicates that there were no marked differences. Only age-, gender-, socioeconomic- and ethnic variations in findings which were explicitly mentioned in a paper will be presented in the synthesis below.

#### 1. Preferences/liking and variety

Most of the papers do not separate taste, preferences, and taste preferences. We distinguish taste and preferences and define preferences as children's choice of some food items over others and taste as a sensory attribute. Across country settings, it is a consistent finding that taste and preferences are important and that children reported a higher preference for fruit than vegetables [16,25,29-39]. *Variety *was identified as an important aspect of preferences in two studies from the US and one from New Zealand. Children who liked a broad variety of fruit and vegetables appeared to be more likely to eat ample amount of these foods. The number of fruit and vegetables liked seemed to differ according to SEP and age [25,32,34].

#### 2. Sensory and physical attributes of fruit and vegetables

Several studies from a range of countries highlighted sensory and physical attributes of fruit and vegetables as both promoters and barriers for fruit and vegetable consumption among children [16,25,27-29,31,33,34,36-47]. The sensory attributes of vegetables were often linked to negative connotations. In one study from the Netherlands, the sensory attributes children considered when evaluating whether they liked or disliked fruit and vegetables differed by age which may reflect different stages of cognitive development [39].

##### Taste/flavour

Many studies concluded that taste is a main reason for not liking fruit and vegetables - especially vegetables [25,27,29,33,34,36,37,39,41,46]. Children reported that *competitive unhealthy food choices *such as sweets and junk foods tasted better [25,34,38,45,48]. However, taste also emerged as a positive outcome expectancy of eating especially fruit in two studies from the US [34,37]. Generally, children appeared to prefer the taste of fruit to vegetables because of the sweet flavour [16,29,32-35,37,38]. Vegetables were associated with unpleasant and negative taste experiences e.g. bitter, sour, taste of nothing, bland, dull, tart, too strong [16,28,31,33,34,36,37,39,44,47]. *Familiarity of taste *and earlier exposure to fruit and vegetables were found to play an important role in children's acceptance of fruit and vegetables in three studies from the US [16,33,36]. Walker et al. (1973) reported that children's distaste for some fruit and vegetables often was more based on prejudice than taste [16]. In another US study, children from youth gardening programmes said that growing and learning more about vegetables made them less prejudiced towards tasting unfamiliar vegetables [43].

The liking and disliking of vegetables was related to *methods of preparation*, in five studies from the US e.g. many schoolchildren did not like cooked vegetables and home prepared vegetables were preferred to those received in school [29,30,34,37,43]. *Addition of a topping/condiment *or another food to enhance the flavour of fruit and especially vegetables was mentioned as a facilitator for fruit and vegetable consumption in a variety of countries [28,29,33,36,39,45].

##### Texture and mouth feel

The texture and mouth feel of fruit and vegetables was reported as another important factor influencing children's fruit and vegetable consumption across country settings. Whereas fruits were perceived as sweet, juicy and fun to eat, vegetables were often linked to negative sensory experiences, e.g. some children expressed that the texture of some vegetables made them feel sick [34,39,46]. Positive aspects of texture that were mentioned were crispy, crunchy and juicy which contributed to children's preference for fresh, raw fruit and vegetables. Negative aspects of texture that were reported across studies were mushy, squishy, slimy, dry, cold, smooshy, gooey, icky, "feels like spittle", too hard, containing seeds, yucky, nasty and related to children's dislike for vegetables, especially cooked vegetables [16,25,29,33,36,38,39,44,46].

##### Food aesthetics (appearance, sight, presentation, appeal)

Stood out as crucial for the children's choice of fruit and vegetables in studies from different country settings [16,29,32,33,38,43-45,49]. Negative aspects of appearance were unripe, mouldy, rotten, wrinkled, "looks horrible", "looks weird", ugly, soggy, and boring. Across studies, children aged 10-18 rejected bruised or imperfect fruit such as brown spots as this was interpreted as possible signs of unsatisfactory taste and texture [16,29,36,40,44-46]. Appearance seemed to be a more important reason for disliking and rejecting vegetables for younger than older children [32,39].

*Colour *was an aspect of appearance mentioned by a couple of US studies. Diversity of colours encouraged children to eat more fruit and vegetables [29]. Children linked colours of fruit to favourable taste expectations whereas they perceived the colour of certain vegetables as unappetizing [16].

##### Smell/odour

In three US studies, a pleasant smell also played a role in the children's fruit and vegetable consumption [16,29,42].

#### 3. Satiety value/hunger satisfaction/filling power

In several studies across geographical settings, children expressed a concern for the satiety value of fruit and vegetables [28,44,45,50]. Children did not think fruit and vegetables were as filling as foods like crisps and chocolate [44]. Another study reported that filling power was an immediate positive outcome expectancy of eating fruit and vegetables [29]. Hunger satisfaction may be a special concern for boys [44].

#### 4. Outcome expectations/expectancies

Outcome expectancies were discussed in several studies from a variety of countries, and could be grouped into positive and negative short and long term expectancies.

##### Short term

Across studies, children were able to list *positive short term outcome expectancies *of eating fruit and vegetables: General health, growth (give you strength, make you stronger, help the body grow and make you taller, more muscular), nutritious and hunger satisfaction (contain vitamins, specific nutrients, good for the eyes and teeth, make you feel full after eating it), cosmetics benefits (looking good, improved body image, improved skin appearance, avoid fatness, remain slender, loose or gain weight, pretty teeth), improve performance and productivity in school work and sports/athletics (fuel for the brain, give energy, refreshing and reviving effect as opposed to unhealthy food that slows down the mind and body), sensory aspects (taste, sweetness, crunchiness, fun to eat) [25,27,29,31-34,36-39,41,42,45,51,52]. *Negative short term expectancies *of eating fruit and vegetables were explicitly discussed in one US study: Make you go to the loo, gas in your stomach, get stuck in your teeth, allergy [29]. In two US studies some of the schoolchildren expressed a concern about the fat and sugar content in vegetables and fruit and the risk of getting hyper and diabetes when eating it [33,40].

##### Long term

One US study reported that children could mention only a few long term outcome expectations (all positive) of eating fruit and vegetables [29]. Three studies from Costa Rica, US and the United Kingdom (UK) suggested that children saw the health damages of not eating fruit and vegetables as a distant concern for adulthood [28,45,50]. Children appear to base their food choices rather on taste, hunger satisfaction, appearance of food, and peer pressure [50]. Other studies reported that children valued the long term outcome expectations (positive expectancy) such as future health as an important reason for eating healthy food [27,29,36,52].

Four studies from the US, New Zealand and Costa Rica suggested that outcome expectancies differed by gender, with boys possibly more concerned with long term disease prevention and general health/fitness aspects and girls with weight loss and weight control [28,32,40,45].

#### 5. Knowledge

##### Dietary/nutritional knowledge

Studies from the US and Costa Rica showed that children knew fruit and vegetables were good for them but they were not specific on the relevance to health [28,36,53]. Some children were able to list specific healthy effects [31,33,36,52]. Hill et al. (1998) reported that dietary knowledge increased by age in New Zealand [32]. Two US studies found that schoolchildren from high SEP neighbourhoods [34] and children who participated in youth gardening programmes could list more specific health benefits of eating fruit and vegetables [36].

##### Knowledge, awareness and acceptability of national recommendations of fruit and vegetable consumption

Four studies included discussion on recommendations. Wind et al. (2005) found low awareness of national recommendations for vegetable consumption among children in the Netherlands and Belgium-Flanders, and many children thought they ate enough fruit and vegetables although hardly any of them ate them every day [38]. According to Campbell et al. (2009) the US schoolchildren in their study knew the recommendations but did not distinguish between fruit and vegetables [30]. In a study among Hmong Americans, children appeared to be more familiar with the definition of a "serving" of fruit and vegetables and the recommendation to eat at least 5 servings of fruit and vegetables daily than their parents [42]. '5-a-day' was perceived as an unreasonably high goal by schoolchildren in the US study by Baranowski et al. (1993) [29].

#### 6. Food categorisation: Perceptions of fruit and vegetables

Ten studies from the US, Australia, the UK, Ireland and the Netherlands examined how children categorise and perceive fruit and vegetables [29,30,34,38,39,44,46-48,52]. Two studies reported that children's perceptions of healthy eating almost invariably included fruit, vegetables and salads [44,52]. Some studies found that children did not classify their food into healthy and unhealthy, rather into liked and disliked [46,47]. Two studies found that children reasoned that if a food tastes bad it must be good for you and the reverse [29,47]. In one study, children (7-12 years old) perceived fruit and vegetables as being appropriate for both children and adults [39] while other studies showed that year 4 and 5 pupils perceived vegetables as being food for grown-ups [34,38]. Some children showed difficulties in deciding which food items belong to the fruit and vegetable group e.g. some children thought that candies containing fruit, soft drinks, lemonade, fruit yoghurt, milk shakes, fruit-flavoured beverages and fruit tea could be defined as fruit or fruit juice or that chips that are based on corn or potatoes could be defined as vegetables [29,30,38]. Other studies found that children did distinguish between fruit and vegetables or perceived salad and vegetables as belonging to different food groups [30,48].

#### 7. Fruit and vegetable preparation skills (behavioural capability)

If children are responsible for preparing their own fruit and vegetable snacks they must be able to peel, cut, chop, grate, core or cook fruit and vegetables. Some US studies suggest that children have limited fruit and vegetable preparation skills and are unsure how to prepare and cook vegetable dishes [25,34]. A study of primarily low SEP children suggested that all children took part in some kind of food preparation [29]. Kirby et al. (1995) found that almost all pupils participated in preparing their own snacks for home or school. Other studies demonstrated sociodemographic variations in preparation skills [32-34,37], e.g. that US children from low SEP sites were responsible for preparing more meals alone than children from middle or high SEP sites [34], that US girls were involved in complex food preparation tasks whereas boys only assisted in simple food preparation task, if any [37], and that older teenagers and children from New Zealand who were living in a one-parent household more often cooked meals on a regular basis than younger teenagers and children living with two parents [32].

There may be national variations in the extent to which parents allow their children to prepare meals. Wind et al. (2005) found that 11-year-olds from the Netherlands were allowed to and liked to prepare fruit and vegetable dishes whereas 11-year-olds from Belgium-Flanders often were not allowed to cut up vegetables themselves or to be involved in preparing the dinner, but that they were allowed to prepare fruit [38]. Similarly some Danish 11-year-olds were not allowed to use sharp knives which prevented them from having pineapples for example if their parents did not cut them up for them [51]. According to Kubik et al. (2005), cooking classes to learn about healthy eating and how to cook cheap food were popular among students in secondary school [54].

#### 8. Convenience and time costs

*Convenience *can be defined as ease of obtaining, preparing, transporting and/or eating fruit and vegetables [45]. Regardless of country setting, lack of convenience emerged as a key barrier to eating fruit and vegetables in several papers [28,31-36,41,44,45,52]. Children were not willing to sacrifice time to eat fruit and vegetables, even when liked [28,32,35,44]. When choosing and purchasing snack food they had a preference for pre-packaged food that was easy to get, to carry and requiring no preparation such as salty snacks, sweets, fast food, and soft drinks [33,42,52]. Fruit and vegetables were in general perceived as inconvenient snack food as they were not instantly available and had to be washed, dried, peeled or cooked before consumed [28,31,32,35,45]. Also fruit and vegetables were inconvenient to transport compared to snacks e.g. fruit gets bruised in your back pack on the way to school [32,38]. However, one study among 13-16-year-olds from New Zealand deviated from this finding and reported that some children saw fruit as a convenient snack food as it did not require preparation as opposed to vegetables. These children also expressed that they expected their parents to cut up fruit for them for snacks [32].

Time appears to be crucial and children accordingly reported to make a trade off between eating healthily and time. Convenience food was preferred in order to be able to sleep longer and to avoid wasting school breaks waiting in a queue [28,35,44,45]. In the Dutch/Belgian-Flemish study by Wind et al. (2005), some participants said they ate fruit during the weekends more often than on school days, because they had more time [38]. Others had less fruit over the weekends because they were too busy doing other things and did not remember to eat fruit.

#### 9. Price and affordability

The importance of affordability was highlighted in ten studies that indicated that price influenced children's purchases and selection of fruit and vegetables in all country settings [25,27,28,32-35,44-46,49,50]. The study by Hill et al. (1998) from New Zealand suggested that children's primary shopping criterion was quantity, i.e. to get as much as possible for their money [32]. Other studies from the US and the UK showed that children were also concerned with quality, satiety value and getting value for money [25,44,50]. Children would not risk wasting money on new food choices or on fruit and vegetables for which there was no guarantee of a pleasant taste as opposed to a chocolate bar which would always taste the same [27,44,50].

In some studies, children said that a trade off between healthy food and hunger satisfaction motivated their food purchase [28,45,50]. They did not think they got value for money if they spent their money on fruit as the price was too high and fruit did not satisfy their hunger. Instead they bought unhealthy food like chips, pastries and fast food that was less expensive and more likely to fill them up. Hill et al. (1998) reported that 13-16 year-olds expected their parents to purchase and provide fruit for them, they would not think of buying it with their own money [32].

Some studies suggested that there were socioeconomic and ethnic variations in the importance of affordability for purchase and consumption of fruit and vegetables [33,34]. In the US, Kirby et al. (1995) found that fruit and vegetables were only perceived as expensive in the lower SES groups [34]. In a study of low income children more Mexican-American children than Caucasian children reported cost as a barrier to fruit and vegetable purchase [33].

#### 10. Situational norms: Appropriateness of time, settings/locations and occasions for eating fruit and vegetables

Children's perceptions of appropriate time, settings and occasions for eating fruit and vegetables influence their opportunities for eating fruit and vegetables.

##### Time

In four studies children stated that dinner was the only appropriate time for eating vegetables [32,34,38,39] which also meant that not having vegetables for dinner was perceived as an important reason for not eating vegetables at all [38]. Fruit, on the other hand was perceived as appropriate to eat at all times of the day in two Dutch studies [38,39]. Zeinstra et al. (2007) noted some age differences e.g. 4-5 year-olds thought that you could only eat fruit for lunch and in the afternoon, whereas 11-12 year-olds thought that you could eat it whenever you felt like eating it [39]. In three studies fruit was usually eaten as a snack [32,34,42]. In only one study from the Netherlands and Belgium-Flanders a few participants mainly from minority groups, said they sometimes ate vegetables (a piece of cucumber, carrot or a tomato) as a snack in between meals [38].

##### Setting/location/occasions

Eating fruit was mainly associated with the home environment [39]. In one study children did not perceive time with friends or TV-watching as appropriate occasions for eating fruit and vegetables and reported they would eat more fruit and vegetables if they could eat it while being together with friends or watching TV [42]. The children in the study by Molaison et al. (2005) mentioned both home, other family members' homes, school and restaurants as locations for eating fruit and vegetables [37]. However, in another study children from all SEP groups agreed that eating out was a special treat and therefore not a time for eating fruit and vegetables [34]. Eating fruit in school was perceived as normal by Dutch and Flemish adolescents whereas eating vegetables in school was perceived as unusual - although some ethnic differences were noted [38].

Dutch and Flemish 11-12-year-olds in two studies reported that fruit was too healthy to serve at birthdays and other celebrations and that the social norm was to eat sweets on such occasions [38,39]. 4-5 year-olds on the other hand reported that fruit and vegetables could be served at parties [39].

#### 11. Availability/exposure to fruit and vegetables at home

Availability of fruit and vegetables at home emerged as an important factor affecting fruit and vegetable consumption in 15 studies from different countries [16,27-30,32-35,37,38,43,45,48,52]. Home availability may reflect socioeconomic and international differences. One example is that vegetables were often not available in Dutch homes whereas they were present in Belgian-Flemish homes [38]. Another example is that children in low income families reported that fruit and vegetables were not available at home [33,37].

##### Variety

Variety was suggested as an important aspect of home availability in studies from the US. Two studies reported that children who were exposed to a wide variety of fruit and vegetables at home, liked and ate a greater variety of fruit and vegetables [16,33]. Lack of variety in the fruit and vegetables available was mentioned as a barrier to fruit and vegetable consumption by children in two studies [27,37]. In one study all child groups expressed having access to a variety of fruit and vegetables at home [48] whereas one study suggested that children in homes in areas of middle to high SEP had access to a larger variety of fresh fruit and vegetables than children from low SEP areas [34]. Furthermore the parents from low SEP regions included canned and frozen fruit and vegetables more often in the family meals [34].

##### Home accessibility, parental facilitation and visibility

Accessibility means whether fruit and vegetables are available in a form and location that make them convenient to eat [55]. The process, where parents increase home accessibility such as by cutting up fruit for their children to eat in between meals is sometimes referred to as parental facilitation [56]. Low accessibility emerged as a key barrier to fruit and vegetable consumption from the US, Denmark, New Zealand and Costa Rica. Fruit and vegetables seemed to be available in most homes but not easily accessible or visible to the children [25,33,48,51].

Children perceived purchase and preparation of fruit and vegetables as an adult task [28,32,33] and reported that they would eat healthier if their parents and adults in school purchased and provided them with healthy food and encouraged them to eat it by cutting up fruit and vegetables or displaying it by having a bowl of fruit on the table [35,42,45].

Two US studies imply that there are socioeconomic and ethnic differences in children's access to fruit and vegetables and parental facilitation [33,34]. For example in one study children from middle and high SEP sites said that their parents cut up fruit and vegetables for them as snacks and left them within easy reach whereas children from low SEP sites were more responsible for preparing their own meals [34].

##### Food rules

The effect of high availability may be limited by the fact that some children are not allowed to eat as much fruit and vegetables as they like or have to ask before they eat fruit and vegetables. For example Belgium-Flanders children often had to ask their parents whether they could take fruit whereas Dutch participants were more likely to be allowed to take fruit themselves whenever they wanted [38]. In a Danish study schoolchildren said that there were no restrictions on consumption of fruit at home whereas they had to ask for permission to take unhealthy food items [51].

##### Home availability of unhealthy competitive food choices

In four studies from the US and Australia, some children discussed availability of unhealthy, competitive food options (visual cues) at home as a barrier to eating fruit and vegetables [31,33,40,52].

#### 12. Availability and exposure to fruit and vegetables in school

Irrespective of country setting, it is a consistent finding across most studies that fruit and vegetables are only available in small quantities in school or not available at all [25-32,35,38,44,46,57].

##### Variety and choice

Children from the US and New Zealand reported they would eat more fruit and vegetables if school offered a larger variety that matched their preferences and served them fresh [26,27,32,57]. Some children said that the choice of fruit in school was limited to canned fruit salad which they did not like or, as for the diabetic children in the study by Gellar et al. (2007) they could not eat it because it was "drenched in sugary syrup" [27,31,32].

##### Quality, appearance and methods of preparation

The children in most studies complained about the quality and appearance of the fruit and vegetables available in school. The fruit items available were often bruised, brown, old-looking or of poor quality e.g. mushy [27,32,35,40,44,45,49]. The vegetables offered at school were cold, mushy, soggy, dry, looked nasty, had an unpleasant taste and were prepared in a non-appealing manner [29,31,44,45,57]. The children in three US studies preferred the way vegetables were prepared at home to the way they were prepared at school [29,34] although some children also thought their parents prepared vegetables in an unappetizing manner [48].

##### Accessibility, visibility, convenience, time costs, and affordability

Regardless of country setting low accessibility in school was highlighted as a barrier to eating fruit and vegetables. Many pupils preferred salad bars, bite-sized or sliced fruit instead of unpeeled or whole fruit [53]. Many children complained about vegetables/salads not being as visible (the children had to ask for them to get them) or promoted as much (e.g. no signs) as unhealthy food items in the school canteen [27,45]. Time was also highlighted as a barrier for example children did not want to waste their precious, but short lunch break waiting in a long queue for healthy food [45,57] and they thought that they had too little time for eating at break time [41]. Some children brought fruit and vegetables from home but not all ate it as transportation ruined the appearance of fruit and vegetables [32,38]. Some pupils thought they would purchase more fruit and vegetables if it was easily accessible in refrigerated vending machines at school [35]. Further, the high price of fruit and vegetables in school was highlighted as a barrier for eating fruit and vegetables in school in studies from Scotland and Australia [46,49].

##### Access to competitive unhealthy food choices

Another barrier to eating fruit and vegetables in school mentioned by children from the US and Costa Rica was the constant and extensive exposure to unhealthy food in the school environment and the lack of access to affordable healthy food options [28,29,31,35,45,53,57].

#### 13. Availability and exposure to fruit and vegetables in neighbourhood/local area

Children in a study from the US said that fruit and vegetables were not available in the grocery stores where their parents purchased food [37]. Seasonality emerged as a barrier to availability of fruit and vegetables in the local area in a study among youth in Minnesota who experienced low access to fresh fruit of high quality in the winter [36]. In two studies children reported that fruit sold nearby was not attractive [32,36]. In three US studies children discussed low availability, variety, visibility, and attractiveness of fruit and vegetables in (fast-food) restaurants as a barrier for intake [34,40,45]. Children only liked eating fruit and vegetables at restaurants with salad bars they could chose their own fruit and vegetables from [34].

Again high *access to competitive unhealthy food choices *emerged as a barrier for fruit and vegetable intake as it appeared to be much more convenient and inexpensive for the children to buy unhealthy foods (fast-food) in the local area than fruit [27,32,34,35,41,45].

#### 14. Parental influence

Some children learned how to eat fruit and vegetables from a family member and found it easier to eat healthily if the entire family did so [31,33] or if they experienced positive social support and motivation from adults to eat healthily [25,30,35,38,42]. However, in one UK study 11-12-year-olds expressed that they did not like being preached to about dietary habits by their parents and as a consequence ate unhealthily to express independence or rebellion [44]. Some studies from the US and Costa Rica also mentioned parents as social influences for eating unhealthy food e.g. by taking the children to fast food restaurants or by having unhealthy eating habits themselves [28,36,48,53]. However, in one US study boy scouts said they were encouraged by their parents to eat fruit and vegetables at restaurants [25].

Hill et al. (1998) found that the family, especially the mother is the primary source of nutritional knowledge among children in New Zealand. In the same study and in a study from Ireland, children said that their parents conveyed mixed messages to them. On the one hand parents tried to limit children's intake of sweets, chocolates, soft drinks and potato crisps, and on the other hand they used the very same unhealthy food items as treats for being good [32,47].

#### 15. Peer influence

Peer influences and peer pressure is a challenging issue in focus group discussions with adolescents. Group dynamics and peer pressure may hinder children from speaking freely about it and also the children may not be conscious of how much they are influenced by their peers [34,36,37,48]. Keim et al. (2001) suggests that in the US, children are very aware of peers' eating behaviours as almost all children in their study knew how much fruit and vegetables their friends ate [33]. In general, across country settings and age groups, peer influences were not perceived as supporting fruit and vegetable consumption, first and foremost because there was a strong peer pressure towards eating unhealthy food [28,31,32,34,37,38,40,46]. Wind et al. (2005) suggest that peers only influence fruit and vegetable consumption among 10-11-year-olds indirectly through promoting higher intake of unhealthy competitive food choices [38], while five studies which covered older age-groups (9-18 years) implied a direct influence as children reported negative comments or being bullied if they brought healthy lunchboxes to school or ate fruit and vegetables in school [25,28,32,40,48,50]. Children did not perceive eating fruit in school as 'cool' behaviour although they ate it at home [41,50].

A few US studies reported positive peer influences towards eating fruit and vegetables and negative peer influences towards eating unhealthy food: Two studies reported that fruit was perceived as acceptable to eat in school but that there was a negative peer influence on eating vegetables in school [25,48]. One study found that children who participated in a youth gardening programme did not perceive any peer pressure towards eating unhealthy food [36]. Some of the diabetic children in the study by Gellar et al. (2007) pointed to their peers as supporting them in eating healthily by encouraging them to avoid unhealthy food to control their blood sugar [31].

Positive and negative peer influences towards eating fruit and vegetables may differ by gender because of the symbolic value of eating fruit and vegetable with respect to image and gender identity [28]. Costa Rican boys were considered effeminate by their peers if they ate healthy food and consequently made it a rule to eat unhealthy food items to prove masculinity and bravery. Among girls, eating healthy foods was considered as a sign of femininity and consequently they often would bring cut up fruit to school. Girls risked being bullied if they did not eat healthily [28]. Also, a study from the US is concerned with the symbolic value of food as a barrier for intake: A girl reported being teased about eating as elderly people because of her high intake of fruit and vegetables which suggests that fruit and vegetable consumption symbolizes old age to some children [40].

Peer interaction may also increase access to unhealthy food in schools [31,46]. Food choice in the playground appeared to be determined by socially acceptable food such as sweets, chocolate, carbonated drinks and crisps rather than fruit and vegetables [41,46]. Two studies from Denmark and Scotland suggested that girls especially valued the social aspects of meals and snacks [46,51]. In the study by Husby et al. (2009) girls often engaged in swapping food with their friends such as exchanging pizza for grapes [51]. Children in other studies said that nobody wanted to swap healthy food such as fruit and they emphasised the importance of bringing food to school that peers wanted to swap to be part of this shared social event [31,46].

#### 16. TV/media influence

Most children had not seen any commercials for fruit and vegetables so media influences were only discussed in relation to promotion of unhealthy competitive food choices. Commercials for unhealthy food and fast food was discussed as a barrier to fruit and vegetable consumption as they triggered a craving towards tasty and inexpensive unhealthy food [25,28,32,36,41,48]. Media influence was not discussed in any of the European studies.

### IV. Sensitivity analysis: results

The potential determinants identified in the low quality studies were generally in agreement with the ones from the high quality studies e.g. home and school availability, convenience, parental modelling and price. However, one contradictory finding was observed: Two low quality studies [27,52] did not observe any gender differences in outcome expectancies, whereas three high quality studies did [28,40,45]. If focus groups mix girls and boys there might be a risk of false agreement due to peer pressure which may conceal gender differences. Further, lack of a gender focus or non-systematic research processes may have hidden gender differences.

The qualitative studies of low quality did contribute with interesting ideas to the synthesis of findings across studies, even though their non-transparent and less reflective research process made it hard to evaluate the credibility of the findings in these studies. For example the study by Kim et al. (2007) adds to the other studies by showing variations in the identified themes appropriate occasions for eating fruit and vegetables (Hmong youth do not perceive being together with friends or TV-watching as appropriate occasions for eating fruit and vegetables) and availability of fruit and vegetables in school (There is a disagreement between the fruit and vegetables children prefer, and what kind of fruit and vegetables are served in school) [42]. Further, two low quality studies indicated that children would eat more if the school increased the variety of fruit and vegetables offered in school [26,27]. Variety was not identified as a potential determinant in any of the middle or high quality studies. Nicklas et al. (1997) was the first qualitative study to focus on high access to unhealthy competitive food choices as a barrier to fruit and vegetable consumption. The study further pointed at visibility as an important dimension of school availability and inconsistency in taste as an important reason for purchasing chocolate, instead of fruit and vegetables [27]. These two themes were not highlighted in other qualitative studies until 2005, where they appeared as influencing factors in the study by McKinley et al. 2005 [44].

If the review had included only the high quality papers most of the key themes would have emerged. However, the themes 'fruit and vegetable preparation skills (behavioural capability)', 'price and affordability', 'parental influence', 'peer influence' and 'knowledge about recommendations for fruit and vegetable intake' had only been superficially described (unsaturated, thin description). Further, the themes 'food rules' and 'variety of fruit and vegetables in school', 'colour and smell of fruit and vegetables' had not emerged as important potential determinants. We had also not captured the range in children's views e.g. for 'satiety value' and 'outcome expectancies'. The study by Baranowski et al. (1993) (quality assessment: medium) added some important dimensions by showing that some children perceived the filling power of fruit as a positive outcome expectancy and that children also associated negative outcome expectancies like for instance allergic reactions and gas build up with eating fruit and vegetables [29]. The high quality studies only highlighted filling power of fruit and vegetables as a barrier to intake [28,44,45].

## Discussion

Thirty-one papers were included in this review and in accordance with earlier reviews of quantitative observational studies [11,13,15,58,59] the present review supports the importance of sociodemographic factors (gender, age/school year, SEP, ethnic background), availability/accessibility of fruit and vegetables at home, parental influences and taste preferences for children's intake of fruit and vegetables. The present synthesis of the results of qualitative studies adds to and enriches the findings in reviews of quantitative studies in five ways (table 3):

**Table 3:** What this review adds

**1.**	Potential determinants for children's fruit and vegetable intake:
	- **Time costs: **trade-off between time and being healthy - **Satiating power: **fruit and vegetables are perceived as less filling than fast food - **Situational norms: **perceptions of appropriateness of time, occasions and settings for eating fruit and vegetables - **Important aspects of availability: **variety, visibility, methods of preparation, quality of fruit and vegetables, access to unhealthy food - **Other important sensory aspects than taste: **appearance, smell, texture - **Price and inconsistency in taste of fruit and vegetables **in comparison with unhealthy food - **Peer influences: **sharing food as a means of socialising, the symbolic value of food for image and gender identity - **School availability: **the importance of variety and being able to make your own food choice, too short breaks for eating fruit and vegetables - **Short term outcome expectancies more important than long term outcome expectations**: Children see long term outcomes as a distant concern of adulthood, they value the immediate benefits or drawbacks of eating fruit and vegetables
**2**.	Extensive information about potential determinants that have only been sparsely investigated in quantitative studies e.g. peer influence, school availability and thereby new input for conceptualisation and operationalisation of these factors
**3**.	Potential mechanisms behind the observed epidemiological associations (or lack of) between personal, social and environmental factors and children's fruit and vegetable intake such as gender and SEP differences e.g. children from high SEP families are exposed to a larger variety of fruit and vegetables at home and thereby may develop a higher preference for a variety of fruit and vegetables which increases their consumption
**4**.	Potential reasons for children's higher intake of fruit compared to vegetables e.g. they perceive fewer time points, occasions and settings as appropriate for eating vegetables than fruit
**5**.	Awareness about the shortage of qualitative studies within this research area from other countries than US

**First of all **the present review has identified some potential determinants for fruit and vegetable intake among adolescents which have only been investigated sparsely in quantitative epidemiologic studies, if at all. These factors are summarized in point one of table 3.

**Secondly**, the qualitative findings enrich the epidemiologic findings in part one of this review and help us understand the variation, scope and implication of the identified determinants. The review of quantitative studies showed that only one out of five studies on outcome expectations found an association between outcome expectations and children's fruit and vegetable intake [11,60]. The qualitative studies suggested that perceived outcome expectations and health benefits of eating fruit and vegetables were not the main concern of children when making food choices rather other aspects such as taste, convenience and sensory appeal mattered.

Further, the review of qualitative studies suggests that cost, appearance, visibility and variety are important dimensions in home availability. None of these dimensions was investigated separately in the studies included in the review of quantitative studies but rather as components of summary scales of 'perceived barriers'. Scales of perceived barriers should be decomposed to investigate the relative importance of items.

The epidemiological evidence from observational studies for an association between availability of fruit and vegetable in schools and pupils' fruit and vegetable consumption is limited [11,58]. Meanwhile a review of intervention studies implies that school fruit and vegetable programmes are effective in increasing fruit and vegetable intake among pupils [61]. The present review of qualitative studies suggests that other aspects of school availability than the mere presence of fruit and vegetables matter such as variety, visibility, quality, texture, cost, convenience, time, access to competitive unhealthy food and methods of preparation. The quantitative studies have focused on parental intake of fruit and vegetables as a promoter of children's own intake [11]. The qualitative studies suggest that it may be worth studying parents' intake of unhealthy food as a barrier for healthy eating habits among children.

**The third contribution **of the present review of qualitative research is that it points at new hypotheses which need to be tested quantitatively. The qualitative evidence suggests that gender roles, peer pressure, the symbolic value of food and different expectancies of the outcomes of eating fruit and vegetables as possible explanations for why girls have a higher or more frequent intake of fruit and vegetables than boys.

Further, a potential chain of causation behind the well-documented social inequality in children's fruit and vegetables intake is suggested. Children from high income families are exposed to a larger variety of fruit and vegetables and as a consequence may develop preferences for a larger variety of fruit and vegetables resulting in a higher intake of fruit and vegetables compared to children from families of low SEP [16,34].

Most epidemiological studies find that young adolescents have a higher or more frequent intake of fruit and vegetables than older adolescents [11]. One possible reason for this suggested by our review is that when children grow older, the extent of parental support for eating fruit and vegetables decreases. Parents expect the older children to prepare fruit and vegetables themselves and stop cutting fruit and vegetables as snacks for them. Another possible explanation for the decline in consumption according to age is that young adolescents' eating habits are primarily under the influence of their parents, whereas the older adolescents are orientated towards their peers. Therefore, older schoolchildren will be more prone to peer pressure towards eating unhealthily. Finally, the teenagers' growth spurt may be another barrier to eating fruit and vegetables as they do not perceive fruit and vegetables as having enough filling power.

**The fourth contribution **of this review is some potential explanations as to why children in most western countries have a higher intake of fruit than vegetables [5,62]. Children's preference for fruit over vegetables appeared to be related to the sensory attributes of fruit and vegetables and to the way vegetables are prepared. Another barrier to vegetable intake may be children's perception of dinner as the only appropriate time and family/home as the only appropriate setting for eating vegetables, whereas fruit can be eaten everywhere at all times of the day. This is in agreement with the quantitative finding that frequency of family meals is associated positively with fruit and vegetable intake [11].

**Finally, as a fifth contribution **the review brings into focus the shortage of published, peer-reviewed qualitative studies from countries outside the US.

No previous systematic review of qualitative studies on children's fruit and vegetable intake is directly comparable to the present review. Jago et al. (2007) found qualitative support for an association between availability of fruit and vegetables and consumption of fruit and vegetables among children and adults in a systematic review of both qualitative and quantitative studies investigating the role of availability [63]. Based on findings from eight qualitative studies they suggested that availability was affected by SEP and location (rural/urban/reservation), but not ethnicity. Our review based on 31 studies is consistent with this [63]. Other systematic reviews among children have focused on determinants of healthy eating, are based on both peer-reviewed papers and grey literature (published and unpublished reports and theses) and have included both quantitative (non-experimental and intervention) and qualitative studies simultaneously [18,64]. Even though their findings are not specific to fruit and vegetable consumption, the facilitators and barriers to healthy eating overlap with many of the identified determinants of fruit and vegetable intake in our review. For example Shepherd et al. (2006) concluded that barriers to healthy eating among 11-16-year-olds were low access to and high cost of healthy food, time considerations and personal preferences for, and easy access to fast food. Facilitators for eating healthily were among other things concerns about appearances and parental support. Furthermore the young people associated fast food with friends and pleasure and valued the ability to choose what they wanted to eat [18]. The 4-10-year-olds in the review by Thomas et al. (2004) valued taste over health, said that "everything that is healthy tastes awful" (page 1011) and did not consider fruit and vegetables as the same kind of food [64]. Further the children perceived their parents as responsible for their personal health e.g. providing the children with fruit [64]. Other reviews in this area are mostly narrative reviews and have not applied systematic review methods to literature search, extraction of data, or quality assessment or been explicit about the methods they have applied [65-68].

### Study limitations and strengths

Qualitative research is multidisciplinary with studies spread over multiple journals and databases. Several papers mention the difficulties in making exhaustive qualitative literature searches due to inconsistent indexing and use of search terms in databases as well as the lack of databases gathering qualitative health research [69-71]. We limited our search to qualitative research by using the search terms anthropology, anthropologic, anthropological, ethnography, ethnographic, ethnographical, qualitative, focus group, focus groups, and grounded theory. We did not use the search term 'interview' as questionnaire surveys are sometimes carried out as interviews. Our search strategy may have excluded qualitative research which has not been indexed correctly by our search terms. Two approaches were used to prevent this. Firstly, the literature search built upon the literature search for quantitative studies for part one of this review. For this literature search we used a less restricted combination of search terms with no requirements for study design ((fruit(s) or vegetables(s)) and ('children or adolescents')) in Medline and PsycINFO. This literature search yielded eight qualitative studies. All of these papers were also identified through our literature search for qualitative studies except a paper published in 1973 which was not indexed as a qualitative study [16]. Secondly, we checked our own records and screened the reference lists for relevant qualitative studies in all full text papers we retrieved for both reviews as well as reference lists of existing reviews. Only one of the 24 additional records we identified through these sources was included in the present review, namely the previous mentioned study from 1973 [16].

We only included peer-reviewed papers in English. These language and publication status restrictions may have posed a risk for publication bias. However, the findings presented in reviews which included grey literature were not in conflict with our conclusions [72,73]. Research published in languages other than English may contribute with important country-specific insight. A few non-English papers from Brazil, Canada, Chile, Germany, and Spain were excluded limiting the variation of settings in the review.

Qualitative findings and interpretations may be more difficult to report in other languages than the language used in the study as translation of quotations into English may result in loss of meaning due to national figures of speech. The fact that 27 out of 31 of the included papers in the review were conducted in English-speaking countries (the US, the UK, Ireland, New Zealand, and Australia) may support this assumption.

We only included findings about children's own views and perceptions of factors influencing their fruit and vegetable consumption and therefore the views of parents and school staff which were expressed in some of the studies included were left out of the summary of findings. Triangulation of sources may enhance the validity of qualitative studies by contributing with more perspectives and dimensions to the phenomenon studied e.g. there may be some constraints on children's intake that they do not perceive as barriers themselves, because other people (parents, school staff, parents of friends) are mainly responsible for increasing children's access to fruit and vegetables. For example parents in two of the studies included said that they expected their children to cut up fruit and vegetables for themselves or ask for help if necessary [25,48] whereas one finding of this review was that children expected their parents to provide them with fruit snacks [28,32,35,42]. The inclusion of both views suggests a discrepancy between children's and parents' perception of their own role and responsibility in relation to increasing accessibility of fruit and vegetables at home.

In the two reviews we have aimed at mapping out all available peer-reviewed quantitative and qualitative evidence on potential determinants of fruit and vegetable consumption among children and adolescents irrespective of country. For the present review we performed a conceptual analysis to synthesise the findings. We analysed potential determinants/themes across studies conducted in different country settings (maximum variation sampling) and deliberately de-contextualised the findings from their original study setting. Any approach which de-contextualize information, e.g. by analysing themes across interviews or across studies instead of focusing on the uniqueness of individuals (case studies) or single studies runs the risk of losing sight of context [69]. To prevent this we could have chosen to make a similar review of qualitative studies within one country setting only (homogeneous sampling). However due to the limited number of qualitative studies identified for this review it would only be meaningful for a few countries such as the US where an abundant number of qualitative studies were conducted.

We evaluated the methodological quality of the papers in two ways. The quantitative approach was a count of quality criteria met. As we did not perceive all quality criteria as equally important for the overall quality, we also conducted an overall qualitative assessment similar to a peer-review process. Our experience was that both methods provided a useful insight into the scientific quality of the included papers. Further, that the quantitative approach constituted a useful basis for the overall qualitative assessment and that the validity of the qualitative approach was improved by obtaining agreement between at least two independent assessors. The qualitative approach is more open for interpretation and dependent on the reviewers' experience with qualitative research.

In the present study we made a comprehensive literature search in 14 electronic databases of biomedical and social science literature which makes it likely that we identified nearly all relevant peer-reviewed studies. Another strength of this study is the systematic and standardised procedures we used to review and evaluate the papers we included. These procedures enriched the review for example by 1) mapping out the variety of views on determinants expressed by children and adolescents across studies, and 2) showing that low quality studies did not detect gender differences in outcome expectations as opposed to studies of high quality. A third strength is that we applied a broad inclusion to obtain the greatest understanding of fruit and vegetable consumption in adolescence [74]. We chose a priori to include all studies that fulfilled the inclusion criteria irrespective of methodological quality. Further, we aimed to present a variety of views and not only those that were mentioned by most children or most focus groups. This approach is in agreement with Fade (2003) who states that "qualitative analysis is not simply a case of counting up the number of times a view is expressed and presenting the most frequently expressed view" (page 144) [22].

### Conclusions and recommendations for research

This review reveals a series of potential determinants of fruit and vegetable intake among children of which our current knowledge is limited (table 3). The prevalence, relative importance and explanatory power of these factors must be assessed in future large scale quantitative studies as well as their interplay with other important determinants such as taste preferences and home availability. The qualitative studies suggest for example, that even when children have a high taste preference for fruit and vegetables they will not eat them if it is not convenient. Even though taste preferences and home availability are necessary prerequisites for children's fruit and vegetable consumption, they may not be sufficient factors. Interactions between home availability, taste preferences, convenience (home accessibility, parental facilitation) and fruit and vegetable consumption should be studied in multivariate analyses.

The concept of availability is often treated as one-dimensional representing whether fruit and vegetables are present at home or in school or not. This review however, suggests that availability is a multidimensional construct and the relative importance of different dimensions of home, school and local area availability such as presence, variety, visibility, quality, texture, cost, convenience, time, access to competitive unhealthy food and methods of preparation should be examined in epidemiological surveys.

Understanding the mechanisms behind the clear sociodemographic differences in fruit and vegetable intake is an important area for future research. The suggested mechanisms from this review should be tested in epidemiological surveys. Such studies may guide the development of subgroup-specific intervention strategies. Future studies should for example clarify the relative importance of different aspects of outcome expectancies of eating fruit and vegetables for consumption among boys and girls and in different age-groups such as positive versus negative expectations, short and long term expectations, and health related versus social outcome expectations (such as expecting parental reward if eating fruit and vegetables).

In the sensitivity analysis we only examined the influence of methodological quality of the included studies on the derived themes. As part of sensitivity analysis, future reviews should also analyse the influence of other study characteristics such as country setting or sociodemographic characteristics of study population.

The review highlights peer influences as important for fruit and vegetable consumption especially among older children and girls. However, peer pressure and peer influences are hard to capture. Qualitative studies and validation studies should aim at conceptualising and operationalising measures of peer support and peer pressure towards eating unhealthily and healthily in order to strengthen future surveys among school-aged children. Multivariate models should examine SEP as a potential effect-modifier for the interplay between exposure to a large variety of fruit and vegetables at home, taste preferences and fruit and vegetable consumption. Furthermore this review confirms that determinants for fruit and vegetable intake should be analysed separately. Children's preference for fruit over vegetables has mainly been explained by taste. This review suggests that other potential barriers for vegetable intake are methods of preparation, texture, appearance and children's ideas of when, where and with whom it is appropriate to eat vegetables. This should be tested quantitatively. Finally, most of the included studies in this review were from the US. Future qualitative studies should explore if the results of this review are transferable to other country settings or if other factors are more important in shaping children's fruit and vegetable consumption there.

### Conclusions and recommendations for practice

This review of qualitative studies emphasises the importance of convenience and the trade off between time and being healthy - issues that could be transferred into product development. Children prefer snacks and chocolate bars, because they are instantly available, can be eaten straight away, do not require any time-consuming preparation, can be carried in your school bag without getting squashed and soggy and because the taste and quality is guaranteed so they do not risk wasting their money on bad quality. These characteristics of snacks can inspire interventions at the family, school and societal level. At the family level, parents should prepare (or assist children in preparing) fruit and vegetables as readily available snacks in between meals (peeled and cut into bite-sized pieces). At the school level, schools could encourage children to eat fruit and vegetables by having a fruit break, where children are allocated time to prepare and eat fruit and vegetables, by offering fresh appealing fruit and vegetables of high quality and variety in the school canteen and refrigerators where the children can store their fruit and vegetables brought from home. At the industrial level, producers should be encouraged to sell fresh (not canned) appealing fruit and vegetable snacks, peeled, cut and ready to eat as a worthy alternative to unhealthy snacks. Super markets should give children visual cues to eat fruit and vegetables by placing the fruit and vegetables near the counter and by advertising them on posters and signs. Furthermore, the availability of less healthy rival food choices should be limited and hidden away from children.

This review has demonstrated the potential of qualitative studies to illustrate the views of children on barriers and facilitators to eating fruit and vegetables. The reviews of qualitative and quantitative studies are not mutually exclusive but complement each other to give a more comprehensive understanding of determinants for children's fruit and vegetable intake. The low number of qualitative studies identified is striking. It is common to do qualitative pilot studies before conducting larger quantitative surveys, but those results are seldom published in scientific journals. However, this review shows that the information gained from qualitative studies is very valuable and researchers should be encouraged to publish results from these studies.

## Competing interests

The authors declare that they have no competing interests.

## Authors' contributions

RK conceived the study, designed and directed the analysis, carried out the literature search data extraction and analysis of the papers included, and drafted the manuscript. MR conceived the study, participated in the design of the analysis and in the selection of papers for inclusion and analysis of the papers included, contributed to the interpretation of data and revised the manuscript critically. JB conceived the study, contributed to the interpretation of data and revised the manuscript critically. KIK conceived the study, contributed to the interpretation of data and revised the manuscript critically. MW contributed to the interpretation of data and revised the manuscript critically. PD conceived the study, participated in the design of the analysis and in the selection of papers for inclusion and analysis of the papers included, contributed to the interpretation of data and revised the manuscript critically. All authors read and approved the final manuscript.

## Supplementary Material

Additional file 1 **Quality assessment tool for systematic review of qualitative studies**. Description of quality indicators used in the quality assessment scheme (table 2). Appendix to Krølner et al. Determinants of fruit and vegetable consumption among children and adolescents: a review of the literature. Part II: qualitative studies. Int J Behav Nutr Phys Act 2011 8:112 doi:10.1186/1479-5868-8-112 (See also table 2 in the paper) Click here for file
